# Supplementary material for: Serum zinc as a biomarker to predict the efficacy of immune checkpoint inhibitors in cancers
Source: PLoS One. 2025 Jul 3;20(7):e0326057. doi: 10.1371/journal.pone.0326057 (PMC12225854; doi:10.1371/journal.pone.0326057)
Supplement: S1 Table — (DOCX) [file pone.0326057.s001.docx]

**Supplementary Table 1:** Usage and dosage of immune checkpoint inhibitors

| **Drug** | **Recommended Dose** | **Frequency** | **Infusion Duration** |
| --- | --- | --- | --- |
| Pembrolizumab | 200 mg or 400 mg | Every 3 or 6 weeks | 30 minutes |
| Nivolumab | 240 mg or 480 mg | Every 2 or 4 weeks | 30 minutes |
| Tislelizumab | 200mg | Every 3 weeks | 30-60 minutes |
| Camrelizumab | 200mg | Every 2 or 3 weeks | 30-60 minutes |
| Sintilimab | 200mg | Every 3 weeks | 30-60 minutes |
| Atezolizumab | 840 mg, 1200 mg, or 1680 mg | Every 2, 3, or 4 weeks | 60 minutes |
| Durvalumab | 10 mg/kg or 1500 mg | Every 2 or 4 weeks | 60 minutes |
