# Supplementary material for: Serum zinc as a biomarker to predict the efficacy of immune checkpoint inhibitors in cancers
Source: PLoS One. 2025 Jul 3;20(7):e0326057. doi: 10.1371/journal.pone.0326057 (PMC12225854; doi:10.1371/journal.pone.0326057)
Supplement: S2 Table — (DOCX) [file pone.0326057.s002.docx]

**Supplementary Table 2:** Setting parameters for the ICP-MS method

| **Operating Conditions** | **Values** |
| --- | --- |
| Extract 2 | -200 [V] |
| Omega Bias | -90 [V] |
| Omega Lens | 7.3 [V] |
| Deflect | 0.2 [V] |
| He flow | 4.3 ml / min |
| OctP RF | 200 [V] |
| Forward Power | 1550 W |
| Nebulizergas flow | 1.07 L / min |
| Nebulizer type | MicroMist |
| Sample introduction | PeriPump |
| Replicates | 3 |
