# Supplementary material for: Serum zinc as a biomarker to predict the efficacy of immune checkpoint inhibitors in cancers
Source: PLoS One. 2025 Jul 3;20(7):e0326057. doi: 10.1371/journal.pone.0326057 (PMC12225854; doi:10.1371/journal.pone.0326057)
Supplement: S3 Table — Detection of baseline levels of 10 selected trace elements. (DOCX) [file pone.0326057.s003.docx]

**Supplementary Table 3:** The serum trace element levels of cancer patients enrolled before the first treatment(T0).

|  | Shapiro-Wilk test：NCB | Shapiro-Wilk test：CBR | P value | Mann-Whitney U/ T value | Median of CBR | Median of NCB |
| --- | --- | --- | --- | --- | --- | --- |
| Fe | 0.9406 | <0.0001 | 0.5155 | 562 | 0.01600 | 0.01650 |
| Mn | <0.0001 | <0.0001 | 0.4894 | 558 | 1.953 | 2.039 |
| Mo | 0.9007 | 0.0057 | 0.2378 | 514 | 1.494 | 1.315 |
| Li | 0.8519 | <0.0001 | 0.2762 | 522.5 | 2.225 | 2.042 |
| Mg | 0.3686 | 0.0979 | 0.1693 | T=1.388 | 0.9498 | 0.9824 |
| Ca | 0.6929 | 0.2368 | 0.1543 | T=1.439 | 2.25 | 2.292 |
| Cu | 0.1725 | 0.0167 | 0.4354 | 550 | 21.23 | 22.66 |
| Zn | 0.1436 | 0.0006 | 0.4224 | 548 | 14.11 | 14.78 |
| As | <0.0001 | <0.0001 | 0.8749 | 606.5 | 0.4650 | 0.4255 |
| Se | 0.0338 | 0.0016 | 0.4275 | 549 | 91.75 | 99.63 |

*Detection of baseline levels of 10 selected trace elements.*
