# Supplementary figures and images for: Serum zinc as a biomarker to predict the efficacy of immune checkpoint inhibitors in cancers
Source: PLoS One. 2025 Jul 3;20(7):e0326057. doi: 10.1371/journal.pone.0326057 (PMC12225854; doi:10.1371/journal.pone.0326057)

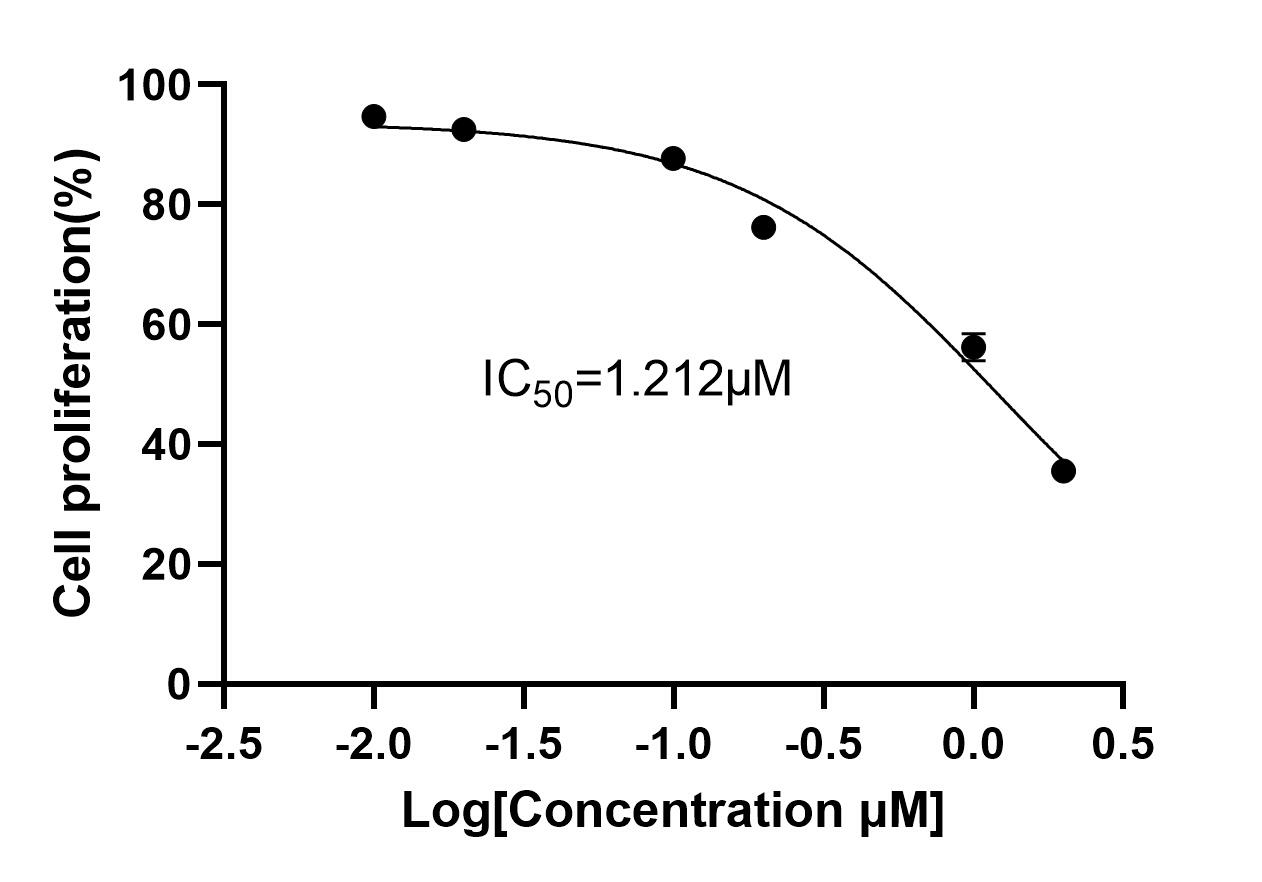

Supplement: S1 Fig — (TIF) [file pone.0326057.s004.tif]
