# Supplementary material for: Serum zinc as a biomarker to predict the efficacy of immune checkpoint inhibitors in cancers
Source: PLoS One. 2025 Jul 3;20(7):e0326057. doi: 10.1371/journal.pone.0326057 (PMC12225854; doi:10.1371/journal.pone.0326057)
Supplement: Attachment — Submitted filename: review1.0.docx [file pone.0326057.s005.docx]

The manuscript under review aims to elucidate the correlation between serum zinc levels and the efficacy of immune checkpoint inhibitors (ICIs) in cancer therapy. The study is distinguished by its retrospective cohort design, employment of advanced assays for serum zinc quantification, exploration of underlying mechanisms through bioinformatics and proteomics analyses, and the innovative proposition that serum zinc could serve as a predictive biomarker.

**Introduction**

**Comment:** The study's background and previous research limitations are presented, yet the specific hypotheses are not clearly articulated at the end. The expected research direction is thus somewhat unclear.

**Comment:** The descriptions of clinical trial results related to immune checkpoint inhibitors could be more concise. Key findings directly relevant to the current study's context should be emphasized, while extraneous details could be omitted.

**Comment:** The transition from discussing the efficacy of immune checkpoint inhibitors to the relationship between zinc ions and immune function lacks a seamless logical connection. A more explicit and well - reasoned explanation is needed to justify the shift in focus and how zinc ions are hypothesized to interact with the immune system in the context of immunotherapy.

**Methods**

**Comment:** The methodology section fails to mention the basis for sample size calculation. Retrospective studies also require sample size estimation.

**Comment:** Despite including 98 patients, the relatively small sample size may not be sufficient to comprehensively explore the complex relationship between serum zinc and immunotherapy efficacy. This could potentially lead to inaccurate or unreliable results. Additionally, the single - center design restricts the patient population's diversity and may introduce selection bias, further compromising the study's external validity.

**Comment:** The lack of a detailed description of the treatment regimens for the various immune checkpoint inhibitors used is a significant omission. Differences in treatment regimens could confound the interpretation of the results, as they may independently contribute to variations in immunotherapy efficacy.

**Comment:** While the discussion acknowledges the presence of uncontrolled confounders such as diet and antioxidant proteins, the methods section should have addressed whether any efforts were made to control for these factors or how they would be accounted for in the analysis.

**Comment:** The selection of specific statistical analysis methods is not adequately explained.

**Results**

**Comment:** Line 210，-Based on this, we selected these four hematologic markers-, four is right?

**Comment:** The hypothesis regarding the lack of significant difference in serum micronutrient levels between the CBR and NCB groups at T0 being a possible result of immunotherapy requires further substantiation. Additional evidence or analyses should be presented to support this claim and rule out other possible explanations.

**Comment:** The omission of P values for the treatment lines group in Table 1 and the excessive footnotes in Table 2 detract from the clarity and readability of the results presentation.

**Comment:** The ROC curves in Figure 2 lack clear labeling. It is unclear whether they represent the training or validation group, and the AUC values are not provided.

**Comment:** The GO and KEGG analysis graphs in Figure 3 contain unexplained terms, which impede the reader's understanding of the results.

**Comment:** In Figure 4, the nomograms (Figures 4A and 4D) lack detailed labeling of the specific meanings and units of measurement for each factor.

**Comment:** The interpretation of the survival analysis results is limited. While the association between serum zinc levels and overall survival is noted, the lack of significant differences in progression - free survival (PFS) requires a more in - depth exploration.

**Comment:** In the multifactorial prediction model section, the description of the model is incomplete. The other factors included in the model (besides zinc and CRP) should be described in detail, and the predictive ability and clinical value of the model need to be more thoroughly evaluated.

**Discussion**

**Comment:** The description of the relationship between serum zinc and immune function lacks a smooth logical flow. The transition between different research perspectives could be improved to enhance the overall coherence of the discussion.

**Comment:** The discussion of the possible mechanisms by which serum zinc affects immunotherapy efficacy is relatively superficial.

**Comment:** The limitations of the study, particularly the single - center design, are not fully explored in the discussion.
