# Supplementary material for: Serum zinc as a biomarker to predict the efficacy of immune checkpoint inhibitors in cancers
Source: PLoS One. 2025 Jul 3;20(7):e0326057. doi: 10.1371/journal.pone.0326057 (PMC12225854; doi:10.1371/journal.pone.0326057)
Supplement: Attachment — Submitted filename: Response to Reviewers.docx [file pone.0326057.s006.docx]

Reviewer #1: The manuscript under review aims to elucidate the correlation between serum zinc levels and the efficacy of immune checkpoint inhibitors (ICIs) in cancer therapy. The study is distinguished by its retrospective cohort design, employment of advanced assays for serum zinc quantification, exploration of underlying mechanisms through bioinformatics and proteomics analyses, and the innovative proposition that serum zinc could serve as a predictive biomarker.

**Responses to Reviewer 1’s Comments**

Thank you for your constructive review. The manuscript has been carefully revised. And the following are responses to the specific comments Introduction.

**Comment:** The study's background and previous research limitations are presented, yet the specific hypotheses are not clearly articulated at the end. The expected research direction is thus somewhat unclear.

**Comment:** The descriptions of clinical trial results related to immune checkpoint inhibitors could be more concise. Key findings directly relevant to the current study's context should be emphasized, while extraneous details could be omitted.

**Comment:** The transition from discussing the efficacy of immune checkpoint inhibitors to the relationship between zinc ions and immune function lacks a seamless logical connection. A more explicit and well - reasoned explanation is needed to justify the shift in focus and how zinc ions are hypothesized to interact with the immune system in the context of immunotherapy.

**Response 1-3:** We have responded by clarifying research hypotheses, streamlining the description of clinical trial results, and strengthening logical transitions. The revised content is located in the introduction section.

Methods

**Comment:** The methodology section fails to mention the basis for sample size calculation. Retrospective studies also require sample size estimation.

**Comment:** Despite including 98 patients, the relatively small sample size may not be sufficient to comprehensively explore the complex relationship between serum zinc and immunotherapy efficacy. This could potentially lead to inaccurate or unreliable results. Additionally, the single - center design restricts the patient population's diversity and may introduce selection bias, further compromising the study's external validity.

**Response1-2:** To assess the adequacy of the sample size, we also referred to similar studies. For instance, Sun et al. observed variations in analyzable data even with a sample size of 40 when investigating tertiary lymphatic structure [1]. In another study evaluating CRP's predictive value for immunotherapy efficacy, only 29 patients were categorized as having a strong response [2], while an intriguing outcome was also reported in a prospective nephritis study involving just 24 samples [3]. These findings were published in the Journal for Immunotherapy of Cancer. Our enrollment primarily consisted of patients receiving first-line immunotherapy combined with chemotherapy, and our sample collection aligns with previous studies that have demonstrated response rates around 20% among immunotherapy recipients [4].

**References**

1. Sun X, Liu W, Sun L, et alMaturation and abundance of tertiary lymphoid structures are associated with the efficacy of neoadjuvant chemoimmunotherapy in resectable non-small cell lung cancerJournal for ImmunoTherapy of Cancer 2022;10:e005531. doi: 10.1136/jitc-2022-005531
2. Klümper N, Saal J, Berner F, et alC reactive protein flare predicts response to checkpoint inhibitor treatment in non-small cell lung cancerJournal for ImmunoTherapy of Cancer 2022;10:e004024. doi: 10.1136/jitc-2021-004024
3. Sise ME, Wang Q, Seethapathy H, et alSoluble and cell-based markers of immune checkpoint inhibitor-associated nephritisJournal for ImmunoTherapy of Cancer 2023;11:e006222. doi: 10.1136/jitc-2022-006222
4. Chen R, Manochakian R, James L, Azzouqa AG, Shi H, Zhang Y, Zhao Y, Zhou K, Lou Y. Emerging therapeutic agents for advanced non-small cell lung cancer. J Hematol Oncol. 2020 May 24;13(1):58. doi: 10.1186/s13045-020-00881-7. PMID: 32448366; PMCID: PMC7245927.

**Comment:** The lack of a detailed description of the treatment regimens for the various immune checkpoint inhibitors used is a significant omission. Differences in treatment regimens could confound the interpretation of the results, as they may independently contribute to variations in immunotherapy efficacy.

**Response:** Thank you for your thoughtful and constructive feedback on our manuscript. We greatly appreciate your observation about the lack of detailed description of the immune checkpoint inhibitor (ICIs) treatment regimen used in our study. At the time of data collection, we counted the drug names and manufacturers of the enrolled patients receiving immune checkpoint inhibitors (ICIs). Its therapeutic dosage and cycle are mainly used according to the drug instructions and the latest guidelines. The following is the specific mode of administration:

| **Drug** | **Recommended Dose** | **Frequency** | **Infusion Duration** |
| --- | --- | --- | --- |
| Pembrolizumab | 200 mg or 400 mg | Every 3 or 6 weeks | 30 minutes |
| Nivolumab | 240 mg or 480 mg | Every 2 or 4 weeks | 30 minutes |
| Tislelizumab | 200mg | Every 3 weeks | 30-60 minutes |
| Camrelizumab | 200mg | Every 2 or 3 weeks | 30-60 minutes |
| Sintilimab | 200mg | Every 3 weeks | 30-60 minutes |
| Atezolizumab | 840 mg, 1200 mg, or 1680 mg | Every 2, 3, or 4 weeks | 60 minutes |
| Durvalumab | 10 mg/kg or 1500 mg | Every 2 or 4 weeks | 60 minutes |

This form will be uploaded with supplementary information.

**Comment:** While the discussion acknowledges the presence of uncontrolled confounders such as diet and antioxidant proteins, the methods section should have addressed whether any efforts were made to control for these factors or how they would be accounted for in the analysis.

**Response:** To control for the potential impact of food or supplement intake on the study outcomes, patients were monitored via telephone and confirmed to have no specific dietary preferences related to zinc or any additional use of zinc supplements. Blood samples were centrifuged within 1 hour after collection to avoid the risk of hemolysis. The absence of red or pink coloration in the serum indicates that our data remained unaffected.

**Comment:** The selection of specific statistical analysis methods is not adequately explained.

**Response:** In this paper, the normality of the grouped data was evaluated, and the choice of T-test, paired T-test or Mann-Whitney U test was made according to the results of the normality test.. Kaplan-Meier regression was used to analyze the relationship between serum trace elements and PFS or OS. To explain the influence of trace elements on the survival time of tumor patients. ROC curves were used to evaluate the accuracy of zinc as a predictor of immunotherapy efficacy and to determine the threshold for serum zinc. Nomograph is derived from univariate and multifactorial Cox regression analyses to establish predictive models for evaluating progression-free survival in immunotherapy. The above statistical methods are commonly used in biomarker studies and have been used in the following articles:

**References**

1. Deng, B., Xiang, J., Liang, Z. & Luo, L. Identification and validation of a ferroptosis-related gene to predict survival outcomes and the immune microenvironment in lung adenocarcinoma. Cancer Cell Int 22, 292, doi:10.1186/s12935-022-02699-4 (2022).
2. Jin, J. et al. A novel ferroptosis-related gene signature for prognostic prediction of patients with lung adenocarcinoma. Aging (Albany NY) 13, 16144-16164, doi:10.18632/aging.203140 (2021).

Results

**Comment:** Line 210，-Based on this, we selected these four hematologic markers-, four is right?

**Response:** Thank you very much for pointing out the relevant error. In fact, we selected these three hematologic markers. We have now revised the corresponding article content.

**Comment:** The hypothesis regarding the lack of significant difference in serum micronutrient levels between the CBR and NCB groups at T0 being a possible result of immunotherapy requires further substantiation. Additional evidence or analyses should be presented to support this claim and rule out other possible explanations.

**Response:** Thank you for your suggestions. Because there are so many factors that affect serum zinc levels, we cannot fully count them. However, we have monitored patients' diet through telephone follow-up. The serum sample should be tested within 1 hour after obtaining to avoid hemolysis affecting the final result. In the follow-up study, we will consider more factors that may affect serum micronutrients, such as endocrine diseases, peritoneal dialysis and X-ray irradiation.

**Comment:** The omission of P values for the treatment lines group in Table 1 and the excessive footnotes in Table 2 detract from the clarity and readability of the results presentation.

**Comment:** The ROC curves in Figure 2 lack clear labeling. It is unclear whether they represent the training or validation group, and the AUC values are not provided.

**Response:** Thank you for your correction. We have readjusted Table 1 to solve the problem that P values appear to be missing due to typography problems. And we removed the footnotes in Table 2.

**Comment:** The GO and KEGG analysis graphs in Figure 3 contain unexplained terms, which impede the reader's understanding of the results.

**Response:** Thanks for your comments, to help readers understand, here are the definitions of the terms included in the figure：

**Comment:** In Figure 4, the nomograms (Figures 4A and 4D) lack detailed labeling of the specific meanings and units of measurement for each factor.

**Response:** We are deeply sorry for our oversight. We have made more detailed annotations of the factors included in the route.

**Comment:** The interpretation of the survival analysis results is limited. While the association between serum zinc levels and overall survival is noted, the lack of significant differences in progression - free survival (PFS) requires a more in - depth exploration.

**Response:** Your comments are very valuable for reference. We attempted to detect the expression level of PD-L1 in patients by immunohistochemistry. However, due to the small tissue of the puncture specimen, some samples could not be detected. We plan to continue to collect pathological samples and conduct cell and animal studies to further elucidate and validate the relationship between serum zinc and immunotherapy.

**Comment:** In the multifactorial prediction model section, the description of the model is incomplete. The other factors included in the model (besides zinc and CRP) should be described in detail, and the predictive ability and clinical value of the model need to be more thoroughly evaluated.

**Response:** Thanks for your comments, we have described the results in more detail.

Discussion

**Comment:** The description of the relationship between serum zinc and immune function lacks a smooth logical flow. The transition between different research perspectives could be improved to enhance the overall coherence of the discussion.

**Comment:** The discussion of the possible mechanisms by which serum zinc affects immunotherapy efficacy is relatively superficial.

**Comment:** The limitations of the study, particularly the single - center design, are not fully explored in the discussion.

**Response1-3:** We have improved the logic fluency of the paper, deepened the mechanism discussion, and modified the research limitation discussion. The detailed changes are listed in the discussion section.

Reviewer #2: An original version of an unconventional approach to the nature of tumors and the possibilities of early diagnostics and drug support of patients, receiving immune checkpoint inhibitors, is presented. Such work reveal the pathogenesis of tumors, allowing us to find new ways to prolong the lives of patients with advanced tumors.

**Responses to Reviewer 2’s Comments**

Thank you for your constructive review.

Reviewer #3: This study first identified serum zinc levels as a novel biomarker for predicting the therapeutic efficacy of immune checkpoint inhibitors (ICIs). Through observing 98 patients with advanced metastatic cancer receiving immunotherapy, using high-precision inductively coupled plasma mass spectrometry (ICP-MS) to measure serum zinc content, the researchers found that patients with serum zinc levels above 14.2μg/L had significantly prolonged overall survival (20.0 months vs 10.0 months). This finding showed a sensitivity of 100% and specificity of 41.86%. Further bioinformatics and proteomics analyses revealed that serum zinc may influence tumor development by regulating cellular DNA replication through MAPK and NF-kB signaling pathways. The research team also established a nomogram model incorporating multiple clinical factors to more accurately predict immunotherapy efficacy. These findings not only provide a new predictive biomarker but also offer new perspectives for understanding the mechanisms of immunotherapy and potential intervention targets for optimizing immunotherapy strategies.

**Responses to Reviewer 3’s Comments**

Thank you for your constructive review. The manuscript has been carefully revised. And the following are responses to the specific comments Introduction.

Major Comments:

**1. Please specify the basis for sample size calculation and statistical power analysis;**

**Response:** To assess the adequacy of the sample size, we also referred to similar studies. For instance, Sun et al. observed variations in analyzable data even with a sample size of 40 when investigating tertiary lymphatic structure [1]. In another study evaluating CRP's predictive value for immunotherapy efficacy, only 29 patients were categorized as having a strong response [2], while an intriguing outcome was also reported in a prospective nephritis study involving just 24 samples [3]. These findings were published in the Journal for Immunotherapy of Cancer. Our enrollment primarily consisted of patients receiving first-line immunotherapy combined with chemotherapy, and our sample collection aligns with previous studies that have demonstrated response rates around 20% among immunotherapy recipients [4].

**References**

1. Sun X, Liu W, Sun L, et alMaturation and abundance of tertiary lymphoid structures are associated with the efficacy of neoadjuvant chemoimmunotherapy in resectable non-small cell lung cancerJournal for ImmunoTherapy of Cancer 2022;10:e005531. doi: 10.1136/jitc-2022-005531
2. Klümper N, Saal J, Berner F, et alC reactive protein flare predicts response to checkpoint inhibitor treatment in non-small cell lung cancerJournal for ImmunoTherapy of Cancer 2022;10:e004024. doi: 10.1136/jitc-2021-004024
3. Sise ME, Wang Q, Seethapathy H, et alSoluble and cell-based markers of immune checkpoint inhibitor-associated nephritisJournal for ImmunoTherapy of Cancer 2023;11:e006222. doi: 10.1136/jitc-2022-006222
4. Chen R, Manochakian R, James L, Azzouqa AG, Shi H, Zhang Y, Zhao Y, Zhou K, Lou Y. Emerging therapeutic agents for advanced non-small cell lung cancer. J Hematol Oncol. 2020 May 24;13(1):58. doi: 10.1186/s13045-020-00881-7. PMID: 32448366; PMCID: PMC7245927.

**2. The background discussion of immunotherapy in the Introduction section is insufficient; suggest incorporating the following references for discussion: PMID: 39225204/35978433/35331128;**

**Response:** Thank you for your modification suggestions. This reference has been included in the introduction to facilitate the discussion of immunotherapy.

**3. Recommend more detailed statistical analysis comparing baseline characteristics between the two groups;**

**Response:** Thank you for your comments. Since this paper mainly discusses whether serum trace elements can predict immune efficacy, we selected serum trace elements of patients before immunotherapy as baseline characteristics analysis. The final results showed no significant difference between the clinical benefit response group (CBR) and the non-clinical benefit group. Therefore, the changes of serum trace elements may be related to immunotherapy. The following are the serum trace elements of the patient before receiving the first immunotherapy. This form is uploaded as supplementary information

|  | Shapiro-Wilk test：NCB | Shapiro-Wilk test：CBR | P value | Mann-Whitney U/ T value | Median of CBR | Median of NCB |
| --- | --- | --- | --- | --- | --- | --- |
| Fe | 0.9406 | <0.0001 | 0.5155 | 562 | 0.01600 | 0.01650 |
| Mn | <0.0001 | <0.0001 | 0.4894 | 558 | 1.953 | 2.039 |
| Mo | 0.9007 | 0.0057 | 0.2378 | 514 | 1.494 | 1.315 |
| Li | 0.8519 | <0.0001 | 0.2762 | 522.5 | 2.225 | 2.042 |
| Mg | 0.3686 | 0.0979 | 0.1693 | T=1.388 | 0.9498 | 0.9824 |
| Ca | 0.6929 | 0.2368 | 0.1543 | T=1.439 | 2.25 | 2.292 |
| Cu | 0.1725 | 0.0167 | 0.4354 | 550 | 21.23 | 22.66 |
| Zn | 0.1436 | 0.0006 | 0.4224 | 548 | 14.11 | 14.78 |
| As | <0.0001 | <0.0001 | 0.8749 | 606.5 | 0.4650 | 0.4255 |
| Se | 0.0338 | 0.0016 | 0.4275 | 549 | 91.75 | 99.63 |

**4. ROC curve analysis should include additional evaluation metrics (e.g., PPV, NPV);**

**Response:** This evaluation metrics has been added to the results.

**5. Suggest including comparative analysis between this study's results and existing related research;**

**Response:** Thank you for your careful review and constructive feedback on our manuscript. Because there are few articles on zinc ion and immunotherapy at this stage, it cannot be analyzed with other relevant articles.

Minor Comment:

**1. Please provide the rationale for establishing inclusion and exclusion criteria;**

**Response:** The collected samples were carefully screened to exclude tumor types that did not conform to the experimental design, resulting in the exclusion of 106 samples including hepatocellular carcinoma, renal clear cell carcinoma, breast cancer, etc. Additionally, 29 patients who experienced treatment interruptions and were lost to follow-up more than twice were also excluded. Ultimately, a total of 98 eligible patients out of the initial pool of 233 patients were identified.

Reviewer #4: This study is highly interesting and provides valuable insights into the analysis of predictive biomarkers for immune checkpoint inhibitors (ICIs). It addresses an important need for non-invasive and cost-effective biomarkers. However, there are a few points that require clarification and further refinement to enhance the overall quality and clarity of the manuscript

**Responses to Reviewer 4’s Comments**

Thank you for your constructive review. The manuscript has been carefully revised. And the following are responses to the specific comments Introduction.

ABSTRACT

**1-To ensure clarity and consistency, it is important to either use the full term "immune checkpoint inhibitors" throughout the manuscript. Avoid using "immunotherapy" in some instances and "ICI" in others.**

**Response:** We have changed "immunotherapy" and "ICI" to "immune checkpoint inhibitors" in the article.

**2-Line 53: In the conclusion, the text seems repetitive. It could be reformulated more concisely.**

**Response:** We have made more concise changes to the paragraph, which are found in the conclusion.

**3-Line 28: The primary aim of the study appears to be the investigation of the correlation between serum zinc levels and ICI effectiveness across different cancer types. Therefore, I recommend removing the phrase regarding the effectiveness of ICIs across different cancer types, as it seems secondary to the main focus of the study.**

**Response:** Thanks for your thoughtful suggestion, we have removed this content.

Introduction

**1-Lines 97-104: This paragraph is duplicated twice: (Interest in the impact of metal ions on immune function has grown markedly in recent years. Evidence is mounting that these ions play a critical role in innate immunity and defense. Genetic variations in ion channels and transporters, identified in individuals with immune deficiencies, further emphasize the importance of ions to immune health).**

**2- You repeat « immune checkpoint inhibitors (ICIs) » multiple times; it would suffice to write simply "ICIs.**

**3- The introduction does not clearly state the objective of the study. It would be helpful to explicitly outline the aim to provide readers with a clear understanding.**

**Response1-3:** We have revised the introduction section to remove more than duplicate parts, and to highlight research highlights to give readers a clearer understanding of the content of this article.

Materiel and methods

**1-Line 168: The term "type of disease" should be replaced with "****histology"**

**Response:** Thanks for your correction, we have replaced the phrase.

**2-Line 182: The paragraph regarding the Nomogram analysis appears incomplete. the discrimination performance of the nomogram, such as C-index, should be included to better assess the model's ability to accurately predict outcomes.**

**Response:** Your suggestions are very valuable and it is really important to check the predictive performance of the nomogram. In this paper, correction curves are used to evaluate the consistency of prediction models. This curve can evaluate the degree of calibration of the model by determining whether the probability of the model's output is consistent with the actual observed probability.

**3-Sometimes"advanced metastatic" is used, while at other times "advanced or metastatic" is used. Please clarify the cancer stages of the patients and ensure consistent terminology throughout the manuscript.**

**Response:** Thank you for your careful review of this article. We have harmonized the terms in the manuscript.

Results

**1-In table 1: Stage I and Stage II patients, which contradicts the description of the cohort as "advanced metastatic." Please clarify whether early-stage patients were included and ensure consistency in the staging description throughout the manuscript.**

**Response:** The sample data finally included in this paper did not include early stage patients. "TNM Stages" in the table is meant to be the stages of T in the TNM stages. But there was a misunderstanding because of our wrong expression. We have used the new classification method and re-performed the statistics.

**2-Line 256: This paragraph contains abbreviations (IC50, CCK8) without providing their full forms.**

**Response:** We are sorry that we didn't provide the full forms. and now the CCK8 and IC50 result analysis charts are uploaded as supplementary information.

**3-Lines 264-267: I observe that while the author focuses on the role of zinc in the response to ICI, the omics analysis appears to diverge from the main topic by emphasizing patients' gene expression without clearly establishing a connection between these genes and zinc levels in patients undergoing ICI treatment.**

**Response:** We analyzed the differential genes of samples with different serum zinc levels, and the signaling pathways involved in these genes, mainly through omics. The results showed that Zn2+ was significantly correlated with MAPK and NF-kB pathways and DNA replication. The NF-kB pathway is involved in PD-L1 expression and affects immunotherapy.

**References:**

1. Vanamee ÉS, Faustman DL. TNFR2: A Novel Target for Cancer Immunotherapy. Trends Mol Med. 2017;23(11):1037-1046. doi:10.1016/j.molmed.2017.09.007
2. Kim, Nayoung et al. “Unveiling the influence of tumor and immune signatures on immune checkpoint therapy in advanced lung cancer.” eLife vol. 13 RP98366. 8 Nov. 2024, doi:10.7554/eLife.98366

**4-Line 271 : In Multi-factor prediction model, The figures provide interesting and visually explicit data, but the text lacks a brief explanation of what they depict. It would be helpful to describe the key elements of the figures in the text and their connection to the study's conclusions.**

**Response:** Thanks for your comments, we have described the results in more detail.

**5- In the methodology, it is stated that "the results were confirmed by flow cytometry," but I could not find the corresponding results in the results section.**

**Response:** We intended to detect the apoptosis rate of A549 cells at different concentrations of zinc ions by flow cytometry and the initial results were significantly different. However, it was later found that there were errors in the result, so the result chart and description were deleted. We are deeply sorry for the inconvenience caused to you by not revising the methodology part in time.

Discussion

**The discussion explores in detail the mechanisms of action and the potential role of zinc in the anti-tumor immune response. However, it seems to overlook concrete studies establishing the relationship between zinc and the therapeutic efficacy of ICIs. It is important for the authors to include such clinical studies, if available, to better support your findings by linking them to existing evidence in the literature, providing a stronger interpretation.**

**Response:** Thanks for your comments, we have revised the discussion section to make it more closely related to the previous content.

Conclusion

**Line 345: The author mentions progression-free survival, but the results only show a correlation with overall survival. Please ensure the text accurately reflects the findings.**

**Response:** We analyzed the overall survival of patients with different serum zinc levels in the results section, and the results showed that a pronounced improvement in overall survival (OS) was noted in patients with higher zinc levels, with a median OS of 20.0 months compared to 10.0 months for those with lower levels.
