# Supplementary material for: Serum zinc as a biomarker to predict the efficacy of immune checkpoint inhibitors in cancers
Source: PLoS One. 2025 Jul 3;20(7):e0326057. doi: 10.1371/journal.pone.0326057 (PMC12225854; doi:10.1371/journal.pone.0326057)
Supplement: Attachment — Submitted filename: review2.0.docx [file pone.0326057.s007.docx]

The revised manuscript has made several improvements, yet there remain areas that necessitate further refinement. Below are specific suggestions for enhancing the manuscript:

1.Sample Size Calculation:

It is recommended to scientifically calculate the required sample size by clearly defining the research objective, selecting the appropriate statistical method, determining the effect size and statistical power, and considering the dropout rate. This approach ensures that the sample size is tailored to the specific needs of the study, rather than relying solely on other literature. Different research objectives, statistical methods, and effect sizes necessitate distinct sample sizes, and thus, a bespoke calculation is crucial.

2.Control of Confounders:

The Methods section should include detailed measures for controlling other potential confounders, such as antioxidant protein levels. If such measures are not feasible, it is essential to discuss the potential influence of these factors on the results in the Discussion section. Addressing potential confounders transparently will strengthen the study's validity and reliability.

3.Survival Analysis Results:

The relationship between progression-free survival (PFS) and serum zinc levels warrants further analysis to elucidate the potential causes.

4.Discussion Section:

The logical coherence between paragraphs in the Discussion section can be optimized to improve the overall flow of the argument.
