# Supplementary material for: Serum zinc as a biomarker to predict the efficacy of immune checkpoint inhibitors in cancers
Source: PLoS One. 2025 Jul 3;20(7):e0326057. doi: 10.1371/journal.pone.0326057 (PMC12225854; doi:10.1371/journal.pone.0326057)
Supplement: Attachment — Submitted filename: Minor comments.docx [file pone.0326057.s008.docx]

**Comments to the Author**
thank you for your revised manuscript.
The modifications made have improved the clarity and quality of the manuscript. However, these are minor suggestions, and overall the case is well-presented and relevant.

1- After the first mention of "immune checkpoint inhibitors (ICIs)", it is not necessary to repeat the full term. You can continue using the acronym "ICIs" throughout the manuscript.

**2-Abstract**

**Line 28:** I suggest rephrasing it to clarify that the study aims to explore the **relationship between serum zinc levels and the effectiveness of ICIs**.

**3-Introduction**

**Line 141 :** Please add **"efficacy of"** before "immune checkpoint inhibition"*.* The revised sentence should read: "This study aims to explore the relationship between zinc levels and the efficacy of immune checkpoint inhibition."

**4-Results**

**Line 319 :** Please consider moving the paragraph about the calibration curve to the beginning of this section « This curve can evaluate the degree of calibration of the model by determining whether the probability of the model's output is consistent with the actual observed probability». It will provide context for how the predictive accuracy of the models was assessed before discussing the nomograms and results.

**5-Discussion**

You have significantly improved the discussion by adding strong arguments to support the results. I only have a few minor suggestions for further refinement.

**Line 332:** advanced or metastatic cancer (Please add metastatic).

**Line 351:** Please remove "immunotherapy" since "ICIs" already refers to it.

**Line 410:** The phrase "In our mouse model of drug-induced lung tumors" seems to reference another author’s work, not the current study. Please, It should be clarified to avoid confusion.

**Line 430:** Please remove the correlation between zinc levels and PFS, as there is no significant difference between low and high zinc levels in PFS (median PFS of 9.0 months for both; p=0.7190, Fig 2B).
